# Targeting the Urokinase-Type Plasminogen Activator Receptor (uPAR) in Human Diseases With a View to Non-invasive Imaging and Therapeutic Intervention

**DOI:** 10.3389/fcell.2021.732015

**Published:** 2021-08-20

**Authors:** Julie Maja Leth, Michael Ploug

**Affiliations:** ^1^Finsen Laboratory, Rigshospitalet, Copenhagen, Denmark; ^2^Biotech Research and Innovation Centre (BRIC), University of Copenhagen, Copenhagen, Denmark

**Keywords:** PET imaging, fluorescence guided surgery, LU domain, uPAR, optical imaging

## Abstract

The interaction between the serine protease urokinase-type plasminogen activator (uPA) and its glycolipid-anchored receptor (uPAR) focalizes plasminogen activation to cell surfaces, thereby regulating extravascular fibrinolysis, cell adhesion, and migration. uPAR belongs to the Ly6/uPAR (LU) gene superfamily and the high-affinity binding site for uPA is assembled by a dynamic association of its three consecutive LU domains. In most human solid cancers, uPAR is expressed at the invasive areas of the tumor-stromal microenvironment. High levels of uPAR in resected tumors or shed to the plasma of cancer patients are robustly associated with poor prognosis and increased risk of relapse and metastasis. Over the years, a plethora of different strategies to inhibit uPA and uPAR function have been designed and investigated *in vitro* and *in vivo* in mouse models, but so far none have been implemented in the clinics. In recent years, uPAR-targeting with the intent of cytotoxic eradication of uPAR-expressing cells have nonetheless gained increasing momentum. Another avenue that is currently being explored is non-invasive imaging with specific uPAR-targeted reporter-molecules containing positron emitting radionuclides or near-infrared (NIR) florescence probes with the overarching aim of being able to: (i) localize disease dissemination using positron emission tomography (PET) and (ii) assist fluorescence guided surgery using optical imaging. In this review, we will discuss these advancements with special emphasis on applications using a small 9-mer peptide antagonist that targets uPAR with high affinity.

## Introduction

The first direct evidence of a high-affinity cellular binding site for the urokinase-type plasminogen activator uPA^1^ (i.e., uPAR) was reported more than 35 years ago (Stoppelli et al., 1985; Vassalli et al., 1985). That discovery represented the final culmination of year’s research to define the “lytic agent that allowed Rous sarcoma virus transformed cells to liquefy the stroma binding cells together” (Fischer, 1946; Dano et al., 1985). We now understand that the “lytic system responsible for this liquefaction” is in fact uPA-mediated plasminogen activation and that the active protease degrading this stroma (insoluble fibrin) is plasmin in a process called fibrinolysis. The seminal discovery of a cellular binding site for uPA uncovered an important hallmark of this system—it provided a mechanism by which cells can focalize and control uPA-mediated plasminogen activation on their cell membrane. The ability to orchestrate the activity of such a powerful proteolytic system on cell surfaces immediately called upon a role in cell migration and extracellular matrix remodeling (Estreicher et al., 1990). The obvious medical implications thereof prompted an intensive research in the structure-function relationships of this system and targeting uPAR in the context of cancer cell invasion and metastasis became a prime objective. The magnitude of that research is illustrated by the fact that a PubMed search on “uPAR and Cancer” yields more than 1,700 entries.

In this review, we will discuss structure-function relationships in the interactions between uPAR and its two principle biological ligands—the serine protease uPA and the provisional matrix protein vitronectin. Although a plethora of potential ligands for uPAR have been proposed over the years and collectively dubbed the “uPAR interactome” (Eden et al., 2011), we will refrain from discussing the molecular properties of these interactions in detail since no solid structural data are available in the form of co-crystal structures. Furthermore, the functional implications of several of these putative uPAR-interactors are either circumstantial or at best indirect (Ferraris et al., 2014). For more information on non-canonical uPAR ligands, their possible role(s) in cell migration and signaling, and their targeting, the reader is referred to the following comprehensive reviews (Blasi and Sidenius, 2010; Smith and Marshall, 2010; Gonias and Hu, 2015; Li Santi et al., 2021; Yuan et al., 2021). We will instead focus on (i) structure-function relationships in uPAR and (ii) recent developments in targeted imaging of uPAR expression using radionuclide probes for positron emission tomography (PET) scanning or near-infrared (NIR) fluorescent probes for optical imaging to assist precision guided cancer surgery.

## Structure of uPAR

In humans, uPAR is encoded by *PLAUR* on chromosome 19q13 and translation of its 7 exons yields a 335 residue long precursor polypeptide. The mature uPAR protein is, however, truncated to 283 residues by posttranslational removal of both N- and C-terminal signal sequences needed for endoplasmic reticulum translocation and glycosyl-phosphatidylinositol (GPI) membrane anchoring, respectively (Ploug et al., 1991). Other modifications include N-linked glycosylation of Asn^52^, Asn^162^, Asn^172^, and Asn^200^ (Ploug et al., 1998b; Gardsvoll et al., 2004) and oxidation of 28 cysteine residues to form 14 disulfide bonds.

### Member of the LU Domain Protein Superfamily

**FIGURE 1:**
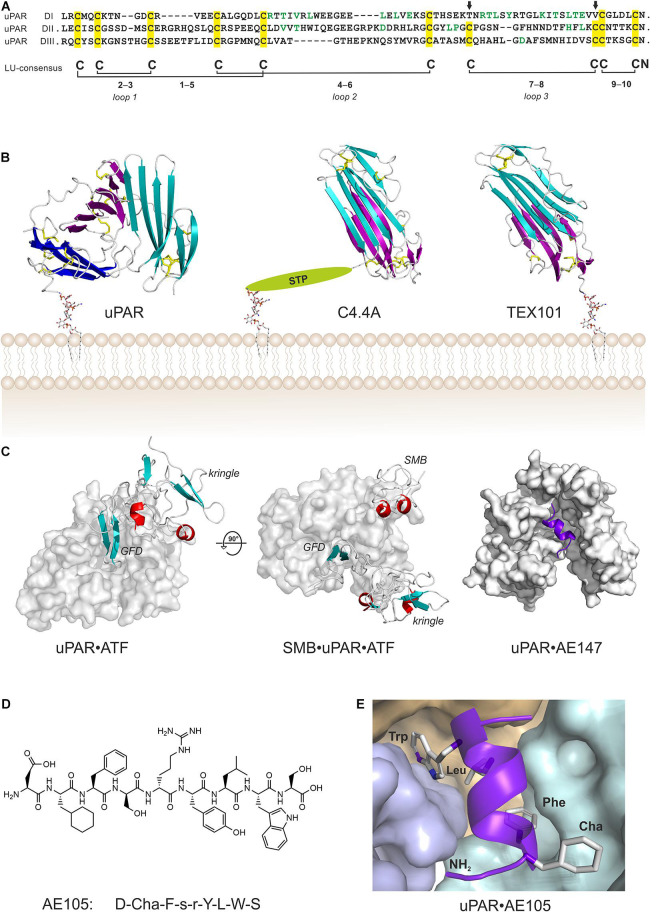
Structure of uPAR in complex with various ligands. **(A)** Sequence alignment of the three LU domains in human uPAR (inter-domain linker regions are omitted for clarity). Cysteine residues are highlighted in yellow and the conserved disulfide bonding are shown. The arrows mark the position of the missing consensus 7–8 LU-disulfide bond in uPAR DI. This pleisiotypic disulfide bond is also absent from the N-terminal LU domain in all other multidomain members of the Ly6/uPAR gene superfamily, but only in the N-terminal domain (Kjaergaard et al., 2008). Residues facing the hydrophobic ligand-binding cavity are shown in green. **(B)** The atomic structures of multi-LU-domain members of the Ly6/uPAR gene superfamily: uPAR with three consecutive LU domains (Xu et al., 2012; Zhao et al., 2015) and C4.4A (Jiang et al., 2020) and TEX101 (Masutani et al., 2020) each with two LU domains. The X-ray structures are shown in a cartoon representation with the β-sheets colored cyan (DI; N-terminal domain), magenta (DII), and blue (DIII) while the disulfide bonds are shown as yellow sticks. The C-terminal of the last LU-domain in uPAR and TEX101 is joined directly with a GPI-anchor moiety, while C4.4A is tethered to the GPI-anchor via a Ser/Thr/Pro–rich linker domain (STP) carrying several O-linked glycans (Hansen et al., 2004). **(C)** Shown are co-crystal structures of uPAR (gray surface representation) in complex with its natural ligand ATF (Huai et al., 2006), and with ATF and SMB (Huai et al., 2008), and in complex with a 13-mer peptide antagonist AE147 (Llinas et al., 2005). **(D)** The chemical structure of the 9-mer peptide AE105 that antagonizes uPA-binding to uPAR with an IC_50_ of 7 nM (Ploug et al., 2001). Cha is (L)-cyclohexylalanine, s is (D)-serine, and r is (D)-arginine. **(E)** The binding pose of AE105 within uPAR’s central ligand-binding cavity. AE105 adopts a short α-helix upon binding (Jorgensen et al., 2004); the binding cavity is assembled by DI (cyan), DII (wheat), and DIII (blue); the hydrophobic side-chains are buried deeply within this cavity (Llinas et al., 2005).

Sequence alignments, limited proteolysis and disulfide bond assignment (Behrendt et al., 1991; Ploug et al., 1993) provided the first evidence that uPAR is a modular protein with three homologous domains related to Ly-6 antigens and snake venom α-neurotoxins (Ploug and Ellis, 1994; Figure 1A). Finally, the intron-exon organization of *PLAUR* reveals that each domain is encoded by separate exon-sets flanked by symmetrical phase-1 introns, which replicates the general construction of genes encoding prototypical single LU domain proteins (Casey et al., 1994; Leth et al., 2019a). Of note, human uPAR deviates from the ancestral LU domain consensus motif inasmuch it contains three consecutive LU domains and that its N-terminal domain lacks one of the five plesiotypic disulfide bond (Figure 1A)—a feature shared among all known mammalian orthologues of uPAR. This is indeed remarkable, as that disulfide bond connecting cysteine 7 and 8 is essential for the correct folding and stability of single LU domain proteins such as SLURP-1 (Adeyo et al., 2015), GPIHBP1 (Beigneux et al., 2015; Kristensen et al., 2021), CD59 (Petranka et al., 1996), and κ-bungarotoxin (Grant et al., 1998). Akin to uPAR, other multidomain members of the LU gene superfamily (e.g., Haldisin, C4.4A, TEX101) also lack this particular disulfide bond, but notably only in their N-terminal LU domain (Kjaergaard et al., 2008; Gårdsvoll et al., 2013; Jiang et al., 2020; Masutani et al., 2020). The evolutionary deletion of the 7–8 disulfide bond in uPAR DI has functional consequences as its reintroduction into recombinant human uPAR impairs both uPA-binding and the dynamic association between uPAR domain DI and uPAR domains DIIDIII in the unoccupied receptor (Mertens et al., 2012; Leth et al., 2019b).

#### Evolution

The plasminogen system developed at the root of vertebrate evolution as an important mediator of fibrin surveillance securing vascular patency in species with a circulatory system. A plasminogen molecule cognate to that found in mammalians arose 550 million years ago (mya) with cyclostomes (i.e., lampreys), while its prime activators, tPA and uPA, appeared 450 mya with jawed-vertebrates (i.e., cartilaginous fishes). The most primitive orthologue known to resemble mammalian uPAR appeared 370 mya at the branch between lungfish and tetrapods; two lungfish genes were found to encode an uPAR-like protein with three consecutive LU domains, each having all 10 consensus cysteine residues (Chana-Muñoz et al., 2019). The unique loss of the 7–8 disulfide bond in the N-terminal LU domain occurred later with the radiation of tetrapods (amphibians, snakes, lizards, turtles, crocodilians, and mammals). Intriguingly, the elimination of that particular disulfide bond co-evolved with the acquisition of an uPA sequence compatible with receptor binding, as deducted from studies on mammals (Lin et al., 2010; Bager et al., 2012; Leth et al., 2019b). Within the avian lineage, the gene encoding uPAR was lost by chromosomal rearrangements, while uPA with a receptor-binding competent growth-factor like domain (GFD) was conserved (Aimes et al., 2003). The inability of a subgroup of avian species to focus uPA-mediated plasminogen activation on their cell surfaces was further exacerbated by the subsequent loss of a gene encoding a prominent plasminogen binding membrane protein (Plg-R_KT_) in the galliform lineage (*Galliformes*), which includes chicken (Sharma et al., 2020).

### Glycolipid Membrane Anchoring

In common with many LU domain proteins, uPAR is tethered to the outer leaflet of the lipid bilayer of the cell membrane via a GPI-anchor. This GPI-anchor is added *en bloc* to Gly^283^ in a transamidase reaction during the posttranslational removal of the C-terminal signal sequence (Ploug et al., 1991; Kinoshita, 2020). This particular mode of membrane tethering provides uPAR with several distinct features such as (i) a prevalent clustering within membrane microdomains or membrane rafts (Varma and Mayor, 1998; Caiolfa et al., 2007; Suzuki et al., 2012), (ii) a mechanism for the specific shedding of uPAR via cleavage of the GPI-anchor by GDE3, a glycerophosphodiester phosphodiesterase (van Veen et al., 2017), and (iii) a deficiency of uPAR on bone-marrow derived blood cells from patients with the hematologic disorder paroxysmal nocturnal hemoglobinuria (Ploug et al., 1992b; Hill et al., 2017). One important corollary of the GPI-anchoring is that uPAR cannot *per se* transmit any signal across the cell membrane and therefore have to rely on indirect signaling pathways, which often complicates the interpretation of causality in molecular terms. The widely accepted model that uPAR-mediated signaling is driven by a promiscuous binding to various integrins (Wei et al., 1996; Smith and Marshall, 2010) has even been challenged by Sidenius and coworkers (Ferraris et al., 2014). They found that the mere binding of uPAR to the matrix protein vitronectin was necessary and sufficient to trigger ligand-independent β1 and β3 integrin signaling. A direct molecular engagement between uPAR and integrins was therefore not required *per se* and the signaling events in that model were relayed by alterations in membrane tension rather than by direct molecular interactions between uPAR and integrins.

### Three-Dimensional Protein Structure

The first atomic structures of uPAR were solved by X-ray crystallography with co-crystals of either a 13-mer antagonist peptide of uPA binding (Llinas et al., 2005) or a receptor binding fragment (ATF) of the natural protease ligand uPA (Barinka et al., 2006; Huai et al., 2006; Lin et al., 2010). In these structures, all LU domains of uPAR assemble via a pseudo-3-fold symmetry to form a large hydrophobic ligand binding cavity, which is delimited by the concave faces of the central β-sheets of the individual LU domains (Figures 1B,C). Of note, this assembly is very different to that found in both C4.4A and TEX101, where the two LU domains assemble with a pseudo-2-fold symmetry by an extensive hydrophobic packing of the concave faces of their central β-sheets (Figure 1B). Construction of uPAR’s multi-domain topology with a flexible assembly of its individual LU domains has important functional consequences—it endows uPAR with cooperativity in uPA- and vitronectin-binding (Madsen et al., 2007; Gardsvoll et al., 2011b; Gardsvoll et al., 2011a; Mertens et al., 2012). This flexibility became first evident by the different uPAR conformations that were trapped in the complexes with the antagonist peptide AE147 and ATF (Figure 1C). Later, biophysical studies on uPAR in solution showed that the N-terminal LU domain (DI) has a high propensity for being detached from uPAR domains DII and DIII resulting in an “open” uPAR conformation, which is driven into a closed and compact conformation by uPA binding (Mertens et al., 2012). Crystalizing uPAR in a closed conformation with an empty ligand binding cavity required that the multi-domain topology was stabilized by either a non-natural disulphide bond between DI and DIII as in uPAR^*H*47*C*–*N*259*C*^ (Gardsvoll et al., 2011b; Xu et al., 2012) or by a monoclonal antibody that bound to the flexible linker region between DI and DII (Zhao et al., 2015). Attempts to determine the structure of “native” uPAR without any stabilizing agents resulted in an electron density map from which a compact structure of uPAR DIIDIII could be determined, but no electron densities corresponding to DI were traceable despite DI was present in the crystals (Liu et al., 2019).

From an evolutionary perspective, the flexible association between uPAR DI and DIIDIII is notable since this association is more prevalent when the pleisotypic 7–8 disulfide bond is absent from DI (Leth et al., 2019b). As discussed previously, the “loss” of this particular disulfide bond in uPAR occurred simultaneously with the acquisition of a receptor binding sequence in uPA. The dynamic detachment of DI provides furthermore a molecular explanation for the uPA- and uPAR-dependent cell adhesion to vitronectin matrices (Wei et al., 1994; Gardsvoll and Ploug, 2007; Madsen et al., 2007). Induction of the closed uPAR conformation by uPA-binding thus increases the affinity between uPAR and vitronectin by assembling a composite binding interface comprising elements of DI, DII, and the linker region connecting these LU domains (Gardsvoll and Ploug, 2007; Huai et al., 2008).

Targeting such a large and dynamic binding interface with small-molecule inhibitors obviously represents a major challenge. Building on structural information on the uPA•uPAR interaction, Meroueh et al. nevertheless succeeded in developing a small compound (IPR-3011) that inhibited AE147 binding to uPAR with an inhibition constant *K*_i_ = 2.4 ± 0.3 μM (Xu et al., 2017). Interestingly, that compound inhibited uPA-binding to the open conformation of uPAR^*wt*^ with a *K*_i_ = 60 ± 5 μM, while it inhibited uPA-binding to the closed conformation of uPAR^*H*47*C*–*N*259*C*^ with a 10-fold improved efficacy *K*_i_ = 6.6 ± 0.4 μM (Xu et al., 2021).

#### The uPA•uPAR Interaction

The uPA•uPAR interaction represents a very high affinity binding with a K_D_ of 19 pM and the complex is long-lived (*k*_off_ = 2 × 10^–4^ s^–1^) as assessed by surface plasmon resonance with purified components (Leth et al., 2019b). This tight binding is replicated on cells with a K_D_ of 55 pM using ^125^I-labeled uPA and isolated monocytes (Nykjaer et al., 1990) and is well aligned with a plasma concentration of 20 pM uPA in healthy donors^2^ (Zeitler and Schuster, 1999). The β-hairpin of the growth factor-like domain (GFD) in uPA represents the key uPAR-binding region, with the hot-spot residues (Tyr^24^, Phe^25^, Ile^28^, and Trp^30^) being buried deeply within the ligand binding cavity (Figure 1C; Huai et al., 2006; Lin et al., 2010). In contrast, more than 15 residues distributed along the surface of uPAR’s ligand binding cavity contribute to the binding affinity for uPA, but none acts as prominent hot spot residues (Gardsvoll et al., 2006). Comparing the atomic structures of unoccupied and uPA-bound uPAR revealed additional flexibility in the multidomain organization. Loop 2 in uPAR DII (residues 130–140) undergoes profound structural shifts to partly cover the entrance of the ligand binding cavity by directly interacting with and covering the β-hairpin of the bound GFD (Barinka et al., 2006; Xu et al., 2012; Zhao et al., 2015). This region, which was dubbed the ligand loading/unloading loop in uPAR, further adds to the complexity of the conformational changes that occurs in the assembly of the LU domains upon uPA binding.

#### The Vitronectin uPAR Interaction

The first evidence that uPAR facilitates cell adhesion to vitronectin-coated surfaces was reported in 1994 (Waltz and Chapman, 1994; Wei et al., 1994). These early studies reported that uPA-binding stimulated the uPAR-mediated cell adhesion to vitronectin. We now know that vitronectin primarily binds uPAR *via* its small somatomedin B (SMB) domain and that this interaction is of relatively weak affinity with a K_D_ of 2 μM (Gardsvoll and Ploug, 2007). In agreement with the potentiation of cell adhesion by uPA-binding, the affinity of the uPAR•SMB interaction increases approximately 3-fold by either uPA-binding or by introducing a non-natural disulfide bond in uPAR^*H*47*C*–*N*259*C*^. Both mechanisms drive uPAR into the closed conformation, lead to robust lamellipodia formations (Gardsvoll et al., 2011a; Gardsvoll et al., 2011b), and increase cell migration on vitronetin-coated matrices (Madsen et al., 2007). Although a 3-fold increase in the affinity of uPAR•uPA for SMB may at first sight appear incremental, its biological impact is likely driven by pronounced avidity effects originating from the binding between uPA–uPAR clusters in lipid rafts and vitronectin molecules embedded in the provisional matrix. Again, inherent flexibility in the assembly of the LU domains in uPAR is required for the allosteric regulation of vitronectin-binding by uPA.

The functional and structural epitope on uPAR for vitronectin-binding is assembled by residues located in uPAR DI (Trp^32^, Arg^58^, Ile^63^), DII (Gln^114^, Arg^116^) and the flexible linker region between DI and DII (Arg^91^, Tyr^92^), and is well separated from the central uPA-binding cavity (Figure 1C; Gardsvoll and Ploug, 2007; Madsen et al., 2007; Huai et al., 2008). This binding interface is dominated by the burial of Arg^91^ within a small cavity in SMB where it forms a strong ionic interaction with Asp^22^ and is flanked by Phe^13^ and Tyr^28^. This binding pose resembles the one found between SMB and Arg^101^ in PAI-1 (Zhou et al., 2003) and the one utilized by the neutralizing monoclonal antibody 8B12 where Asp^99^ forms ionic interactions with Arg^89^ and Arg^91^ in uPAR (Zhao et al., 2015). One puzzling observation is that uPAR-binding of vitronectin completely buries a linear sequence in the linker region between uPAR DI and uPAR DII (–Ser^88^–Arg^89^–Ser^90^–Arg^91^–Tyr^92^–), which has been implicated in uPAR-mediated chemotaxis, directional cell migration, and angiogenesis *via* the formyl-peptide receptor type 1 (Resnati et al., 2002; Bifulco et al., 2010; Minopoli et al., 2019).

## uPAR Biology

Baseline expression levels of uPAR are generally low in most homeostatic tissues and its scattered expression is primarily confined to bone-marrow derived white blood cells, pulmonary alveoli, glomeruli, and a few quiescent endothelial cells (Solberg et al., 2001). In the gastrointestinal tract, uPAR is likewise absent from most epithelial compartments, except from the antrum and the transitional cells of the squamo-columnar junction in mice (Alpízar-Alpízar et al., 2020). In contrast, uPAR expression is upregulated in many tissues undergoing active remodeling such as (i) the leading edge keratinocytes during re-epithelialization in wound healing (Romer et al., 1994), (ii) the invasive extravillous trophoblasts during early embryo implantation (Multhaupt et al., 1994; Plaisier et al., 2008), and (iii) the regressing glandular tissue during mammary gland involution (Solberg et al., 2001).

Despite an elevated uPAR expression during tissue remodeling, congenital uPAR deficiency is clearly not detrimental to development, survival, or reproduction as *Plaur^–/–^* mice are fertile with no overt early-onset phenotypes (Bugge et al., 1995b, 1996a). The mild phenotypes of uPAR deficiency is very different from those associated with plasminogen deficiency. Humans with type I plasminogen deficiency/severe hypoplasminogenemia (Schott et al., 1998) and *Plg^–/–^* mice (Bugge et al., 1995a; Romer et al., 1996; Lund et al., 2000) both display multiple adverse clinical manifestations due to a progressive extravascular fibrin deposition. In long-term studies, mouse strains that were unable to focus plasminogen activation on their cell surfaces due to wholesale gene ablations (*Plaur^––/––^* or *Plau^––/––^*) or gene replacement with an uPAR binding–incompetent uPA-variant (*Plau*^*GFDhu/GFDhu*^)^3^ all developed chronic hepatic inflammation associated with an impaired fibrin surveillance (Connolly et al., 2010). Cooperating this role in extravascular fibrinolysis, uPA and uPAR expressing endothelial cells and macrophages were found to line the surfaces of fibrinoid deposits in the placenta (Pierleoni et al., 2003). In humans, genetic studies find no association between single missense variants in *PLAUR* and a robust risk for disease predisposition, except for a few publications where *PLAUR* variants located outside the protein coding regions were correlated to vascular complications in patients with systemic sclerosis (Manetti et al., 2011) and to a decline in pulmonary function in asthma patients (Barton et al., 2009). Collectively, these studies indicate (i) that in healthy individuals uPAR is not critical to the function of vital tissues and (ii) that uPAR assists in long-term fibrin surveillance alleviating chronic inflammation provoked by fibrin deposition. The pathogenesis associated with impaired plasminogen activation is primarily driven by an excessive fibrin deposition since the severe pleiotropic phenotypes of *Plg^–/–^* mice were more or less absent in double-deficient *Plg^–/–^ Fib^–/–^* mice (Bugge et al., 1996b).

### Role in Normal Physiology

#### Cell Surface Associated Plasminogen Activation

**FIGURE 2:**
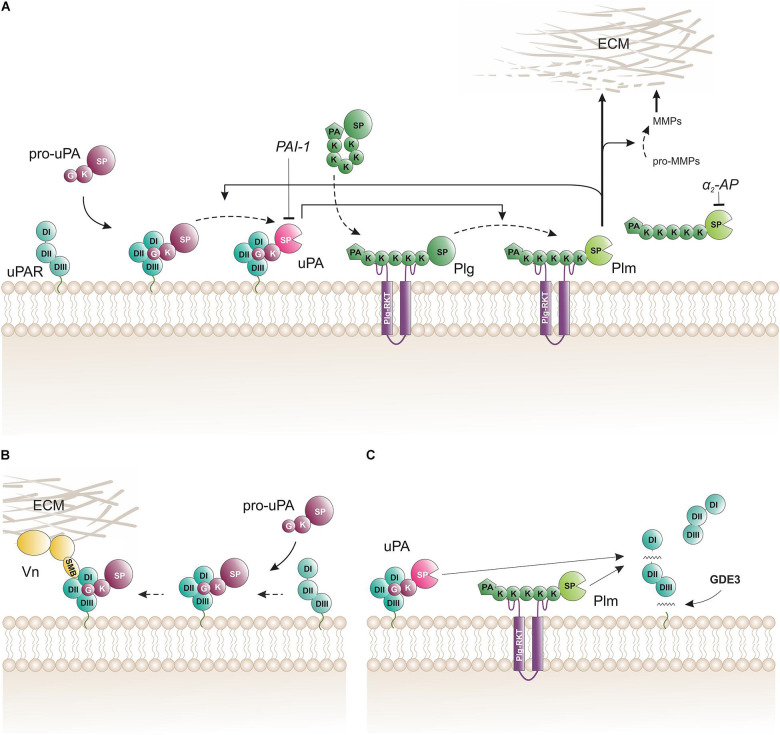
Biochemical pathways for proteolytic function(s) of uPAR on the cell surface. **(A)** The membrane template for an amplified cell surface associated generation of plasmin activity by reciprocal zymogen activation of pro-uPA and plasminogen (Plg). Fluid-phase propagation of plasmin (Plm) activity is prevented by the inhibition of dissociated Plm by its cognate inhibitor α_2_-antiplasmin (α_2_-AP). Regulation of the amplification by this template is also secured by the irreversible inhibition of uPA by a dedicated serpin, the plasminogen activator inhibitor type-1 (PAI-1). **(B)** Engagement of pro-uPA•uPAR complexes in cell adhesion to vitronectin (Vn) in the provisional matrix. Interactions between the closed conformation of uPAR (induced by pro-uPA binding) and the somatomedin B domain (SMB) of vitronectin tethers the cell to the vitronectin-rich extracellular matrix (ECM) and this presumably causes indirect activation of integrins and relay cell signaling altering cell adhesion and migration (Smith and Marshall, 2010; Ferraris et al., 2014). This uPAR-dependent adhesion is weakened when the cell surface template becomes activated since the proteolytic activity of the generated membrane-bound uPA and Plm will release the SMB domain from vitronectin thus breaking the tether between the cell surface and the matrix (De Lorenzi et al., 2016). **(C)** Membrane shedding of soluble uPAR variants. The glycerophosphodiester phosphodiesterase GDE3 may shed intact uPAR or uPAR DIIDIII by cleaving their GPI-anchor (van Veen et al., 2017). Proteolytic activity from membrane-bound uPA or Plm efficiently cleaves uPAR in the flexible linker region between DI and DII thus releasing uPAR DI. *K*, kringle domain; *G*, growth-factor like domain; *SP*, serine protease domain; *PA*, apple domain; *SMB*, somatomedin B domain; *DI*, *DII*, *DIII*, LU domains in uPAR.

*In vitro* studies with purified proteins and cultured cells provided an outline of the biochemical pathway orchestrating cell surface associated plasminogen activation (Figure 2A). The hallmark of this pathway is the separate docking of two zymogens on the cell surface by: (i) a specific and high-affinity interaction between uPAR and pro-uPA (K_D_ ∼ 20 pM); and (ii) a low-affinity binding of plasminogen to an array of broadly distributed membrane proteins with C-terminal lysine residues (K_D_ ∼ 1 μM), including Plg-R_KT_ (Miles et al., 2014). Of note, Plg-R_KT_ colocalizes with uPAR on cell surfaces (Andronicos et al., 2010). The concomitant binding of pro-uPA and of plasminogen to cell surfaces provides a template for enhanced plasminogen activation due to an increased efficiency of the reciprocal activation of the two zymogens i.e., pro-uPA activation by plasmin and plasminogen activation by receptor-bound uPA thus forming a positive feedback loop. This arrangement lowers the *Km* for plasminogen activation from 25 μM in solution to 0.7 μM for cell-bound reactants, which is well aligned with a plasma concentration of 2 μM plasminogen (Ellis et al., 1991). Of note, cell-bound plasmin is refractory to inhibition by α_2_-antiplasmin. This differential sensitivity to α_2_-antiplasmin mediated inhibition provides a further drive toward a focal confinement of plasminogen activation on cell surfaces.

The inherent power of arming cells with such a potent protease system on their cell-surface is clearly demonstrated *in vivo* by the severe cutaneous phenotypes (phemphigoid lesions) that develop in bitransgenic mice, in which *Plaur* and *Plau* transcription in the skin is controlled by the keratin 5 promoter (Zhou et al., 2000). Assembly of a functional cell-surface template with plasminogen and uPAR-bound pro-uPA is required to drive this dermal pathogenesis, since neither of the single transgenic mice nor the bitransgenic mouse crossed into a Plg*^–/–^*background develop these lesions (Bolon et al., 2004). The necessity for an intact and functional template to activate plasminogen is also found under non-pathological conditions, since mice with single deficiencies in either *Plaur*, *Plau*, or *Plg* are resistant to an engineered anthrax toxin that requires proteolytic activation by surface bound uPA (Liu et al., 2003). That dependency is further underscored by the lack of toxicity in the *Plau*^*GFDhu/GFDhu*^ mouse strain, which produces a catalytic proficient pro-uPA that fails to bind uPAR (Connolly et al., 2010). It is therefore beyond any reasonable doubts that cell-surface plasminogen activation driven by the assembly of pro-uPA•uPAR complexes is operational *in vivo*. The mild overt phenotypes of *Plaur^–/–^* mice and their late-onset is most likely a consequence of functional redundancy, since mice with combined deficiencies for uPAR/tPA or for uPA/tPA (the two prime plasminogen activators) exhibit exacerbated hepatic fibrin deposition compared to *Plaur^–/–^* mice (Bugge et al., 1996a).

The membrane-bound template for pro-uPA and plasminogen activation is also implicated in the proteolytic activation of an oncogenic transmembrane receptor denoted CUB domain containing protein 1 (CDCP1) (Kryza et al., 2021). A proteomic search in three different cancer cell lines with a protease-reactive warhead build on CDCP1 sequences, identified uPA and plasmin as the lead candidates for cleaving CDCP1 at Arg^368^ or Lys^369^. Proteolytic processing of CDCP1 at these sites leads to potentiation of its pro-metastatic effect. Subsequent mechanistic studies demonstrated that uPA needed to be tethered on the cell surface via uPAR binding to promote the cleavage of CDCP1 (Kryza et al., 2021).

#### uPAR in Vitronectin-Dependent Cell Adhesion

While the impact of uPAR and uPA•uPAR complexes on cell adhesion and migration is well documented *in vitro* by a plethora of different cell culture experiments (Madsen et al., 2007; Petzinger et al., 2007; Salasznyk et al., 2007; Smith and Marshall, 2010; De Lorenzi et al., 2016), their impact (if any) under normal physiologic conditions *in vivo* is less clear. Like *Plaur^–/–^* mice, *Vtn^–/–^* mice have no overt phenotypes that would support a vital role for vitronectin in cell adhesion and migration during normal development (Zheng et al., 1995). Nonetheless, one study found that *Vtn^–/–^* mice had slightly slower dermal wound healing, reduced dermal microvessel density, and focal sites with delayed hemorrhage (Jang et al., 2000). The origin of this mild phenotype was presumably endorsed by an unbalanced activity of tPA and uPA in the wound field allegedly due to a faster latency transition of their shared inhibitor PAI-1 in the absence of vitronectin. An intriguing rendezvous between uPAR-dependent cell adhesion and cell-surface associated plasminogen activation that is mediated by uPA•uPAR complexes provides a possible regulatory mechanism to control the adhesion between uPAR-positive cells and vitronectin-rich provisional matrices. This interplay involves three sequential steps: (i) focal adhesion initiated by the binding between pro-uPA–uPAR complexes on the cell surface and SMB in vitronectin that is deposited as part of the provisional extracellular matrix (Figure 2B; Madsen et al., 2007), (ii) manifest cell adhesion and migration after relay of signals *via* indirect coupling to integrins, receptor tyrosin kinases, or G-protein-coupled receptors (Smith and Marshall, 2010), and finally (iii) attenuation of cell adhesion and migration by activation of pro-uPA to uPA (De Lorenzi et al., 2016). Of note, zymogen activation within the pro-uPA•uPAR•plasminogen template thus provides a negative feedback loop on cell adhesion by promoting the cleavage of either uPAR in the linker region between DI and DII (Figure 2C) or at the R↓GD-motif in the linker to the SMB domain (Hoyer-Hansen et al., 1992, 1997; De Lorenzi et al., 2016). Both cleavages dismantle the ternary uPA•uPAR•vitronectin complex and are mediated by either uPAR-bound uPA or by cell surface associated plasmin.

### uPAR in Pathophysiology

In general, pathologies associated with chronic inflammation, such as rheumatoid arthritis, cancer, and Crohn’s disease, display elevated uPAR expression levels at their lesion sites primarily due to infiltrating immune cells. While evidence for a direct causal effect of uPAR-expression on disease progression often remains uncertain (Almholt et al., 2015), elevated uPAR levels in resected lesions or shed into the circulation are strong and robust surrogate biomarkers of disease severity. Most observational studies find an inverse correlation between elevated plasma levels of soluble uPAR and patient performance and survival (Lund et al., 2011; Madunic, 2018).

#### Soluble uPAR as a Surrogate Biomarker for Cancer Progression

Building on the seminal discoveries that plasma levels of uPA (Duffy et al., 1990) and soluble uPAR (Stephens et al., 1999) are robust biomarkers for adverse disease progression, accumulating observational studies continue to underscore that high uPA and/or high uPAR levels predict poor prognosis for patients with solid tumors (Grunnet et al., 2014; Dohn et al., 2015; Brungs et al., 2017; Loosen et al., 2018, 2019; Lu et al., 2018; Madunic, 2018). The source(s) of shed soluble uPAR is not always known, but the activated tumor-stromal microenvironment is a possible culprit from which uPAR may be released by proteases and/or hydrolases cleaving the GPI-anchor e.g., GDE3 (Figure 2C). The linker region between uPAR DI and DII is highly susceptible to proteolytic cleavage by e.g., uPA or plasmin, which releases uPAR DI from the cell surface (Hoyer-Hansen et al., 1992). Time-resolved fluorescence immunoassays with high sensitivity and specificity were therefore developed and validated for quantification of intact uPAR and its cleavage products in plasma (Piironen et al., 2004; Thurison et al., 2010). The baseline expression levels of uPAR, uPAR DIIDIII, and uPAR DI in healthy subjects are low; 36.5, 13.9, and 18.6 pM, respectively (Thurison et al., 2015). Subsequent studies showed that these soluble uPAR fragments were independent prognostic biomarkers in pre- and post-operative plasma samples from patients with colorectal cancer (Rolff et al., 2019). For more detailed information on uPAR expression and cancer dissemination the reader is referred to the following reviews (Andreasen et al., 2000; Romer et al., 2004; Allgayer, 2010; Kriegbaum et al., 2011; Madunic, 2018; Li Santi et al., 2021).

#### Paroxysmal Nocturnal Hemoglobinuria

The etiology of a rare hematological disorder termed paroxysmal nocturnal hemoglobinuria (PNH) is the clonal expansion of hematopoietic stem cells carrying somatic loss-of-function mutations in *PIGA*, which encodes an enzyme pivotal for the biosynthesis of GPI-anchors (Hill et al., 2017; Kinoshita, 2020). The overarching hallmark of the PNH syndrome is that affected blood cells fail to express GPI-anchored proteins on their cell membranes and instead secrete a soluble truncated variant lacking the C-terminal signal sequence for GPI-anchoring. Accordingly, monocytes and neutrophils affected by PNH are deficient in membrane-tethered uPAR (Ploug et al., 1992b) and they secrete a soluble uPAR (Ploug et al., 1992a) leading to sustained elevated plasma levels of soluble uPAR with an average plasma concentration of 120 pM (Ronne et al., 1995; Sloand et al., 2008). Due to the pleiotropic effects of *PIGA* deficiency, the molecular causality underlying the clinic manifestations of PNH are often complex and incompletely delineated. Notwithstanding this uncertainty, treatment with a neutralizing anti-C5 antibody (eculizumab) alleviates all of the major adverse clinical complications in PNH i.e., intravascular hemolysis, venous thrombosis, renal dysfunction, and pulmonary hypertension (Hill et al., 2017). It is therefore likely that the principal driver of these pathogenic complications in PNH is intravascular hemolysis due to CD59 deficiency and that eculizumab treatment compensates for the impaired complement regulation. Secondary to episodes of intravascular hemolysis, excessive renal resorption of hemoglobin dimers accompanied by heme cytotoxicity may thus drive the development of acute and chronic kidney disease (Kokoris et al., 2018).

#### uPAR in Kidney Disease

Soluble uPAR levels in patients with idiopathic focal and segmental glomerulosclerosis (FSGS) has attracted a lot of attention after Reiser et al., proposed that soluble uPAR was a prime candidate for the elusive serum permeability factor responsible for recurrent FSGS after kidney transplantations (Wei et al., 2008, 2011). Among other findings, they based their proposition on (i) that the elevated plasma levels of soluble uPAR in primary and recurrent FSGS were independent of the estimated glomerular filtration rates (eGFR) and (ii) that glomerular uPAR deposits activated podocyte α*_v_*β_3_ and thereby drove foot process effacement leading to manifest proteinuric glomerular disease. Although this hypothesis initially gained some support, it nevertheless had some inherent weaknesses and it remains highly controversial (Konigshausen and Sellin, 2016; Kronbichler et al., 2016; Saleem, 2018). First, it was questioned whether the increased levels of soluble uPAR in FSGS patients actually were unrelated to decreased eGFR (Meijers et al., 2014; Musetti et al., 2015). Second, the finding that elevated levels of circulating soluble uPAR should be a pathogenic factor *per se* causing proteinuria and onset of FSGS in mouse model systems could not be reproduced by several independent laboratories (Cathelin et al., 2014; Spinale et al., 2015; Harel et al., 2020). One independent group (Alfano et al., 2015) did, however, report that a single *iv* injection of 20 μg full-length soluble mouse uPAR induced proteinuria after 24 h, but these studies were conducted in *Plaur^–/–^* mice and therefore have little bearings on clinical FSGS—in common with the original studies by Reiser et al. Third, it is difficult to reconcile a causative pathogenic role of soluble uPAR in humans with the fact that patients with PNH have life-long and marked elevations of their plasma uPAR levels, but the renal dysfunction found in PNH patients can be treated with eculizumab showing that it is secondary to complement-mediated hemolysis (Kokoris et al., 2018).

Adding to the complexity in the tale of the pathogenic effects of soluble uPAR in FSGS, Reiser et al., reported that an alternative splice variant of mouse uPAR was the more potent nephrotoxin compared to the conventional soluble uPAR with intact LU-domains (Wei et al., 2011, 2019). Building on our extensive knowledge from protein structure-function analyses on uPAR, this proposal is very surprising. The alleged protein product from this alternative splice variant would thus comprise the N-terminal LU domain, the linker region and only half of the second LU domain and contain an uneven number of cysteine residues. Generally, the folding of LU domain proteins are very sensitive to mutations of their consensus cysteine residues leading to misfolded and aggregated proteins (Leth et al., 2019a). Notwithstanding these concerns, Reiser et al., reported a low resolution (17 Å) structure of this particular uPAR splice variant based on single particle reconstructions from electron microscopy images (Wei et al., 2019). Whether this structure actually represents the elusive uPAR splice variant in question remains nonetheless unclear to us, since their protein preparations also contained at least one dominating protein contamination of 50–80 kDa (as judged by their SDS-PAGE under reducing conditions). Furthermore, their native PAGE reveals a lot of protein aggregation as would be predicted for this construct from our general knowledge of LU domain protein stability (Leth et al., 2019a). As a consequence, we still consider the existence of such a *properly folded* truncated uPAR splice variant for highly speculative both *in vitro* and *in vivo*.

#### uPAR in Rheumatoid Arthritis

Chronic rheumatoid arthritis (RA) is a progressive inflammatory disease that ultimately leads to irreversible joint destruction by an invading pannus causing cartilage degradation and bone erosion. Studies on biopsies from arthritic lesions in RA patients by *in situ* zymography revealed pronounced elevations in uPA activity in the hyperproliferative synovial lining (Busso et al., 1997). Accordingly, these lesions express markedly elevated levels of both uPA and uPAR by infiltrating neutrophils, macrophages, and fibroblast-like cells in the inflamed RA synovium (Almholt et al., 2018) and increased plasma levels of soluble uPAR correlate to disease activity (Slot et al., 1999; Enocsson et al., 2021).

Several mouse models have been developed as experimental surrogates of rheumatoid arthritis in humans; please consult Buckley et al. (2019) for a comprehensive review. One widely used mouse model that mimics non-septic systemic polyarthritis in humans is the autoimmune collagen type-II induced arthritis model (CIA). Elegant genetic dissections, provided strong evidence for the causal role of uPA-mediated plasminogen activation in driving inflammatory CIA. First, *Plg^–/–^* mice were shown to be resilient to CIA and to a cocktail of mouse monoclonal anti-collagen type-II auto-antibodies, but daily administration of purified human plasminogen restored their sensitivity to these treatments (Li et al., 2005). Second, *Plau^–/–^* and *Plaur^–/–^* mice showed none or only very mild symptoms of arthritis after being challenged with collagen type-II (Thornton et al., 2017). In all three genotypes, the adaptive immune response was intact and mounted a similar response toward collagen type-II as did littermate wild-type control mice. With reciprocal bone marrow transplantations, the impact of uPAR to disease incidence and severity was traced to cells originating from the bone-marrow compartment with inflammatory monocyte/macrophages being the likely culprits (Thornton et al., 2017).

Prompted by the beneficial impact of *Plau^–/–^* and *Plaur^–/–^* on the incident and severity of CIA, Almholt et al. (2018) used neutralizing murine monoclonal antibodies toward mouse uPA (mU1) or mouse uPAR (mR1) to treat progression of arthritis in the CIA model. Aligned with the genetic data, inhibition of uPA catalytic activity with mU1 markedly alleviated the pathology and the adverse disease progression of CIA. Unexpectedly, treatment with mR1 showed no beneficial effect, which is unexpected given the beneficial effects observed in either (i) *Plaur^–/–^* mice (Thornton et al., 2017) or (ii) in a gene therapy approach competing the uPA•uPAR interaction with a hybrid molecule between human serum albumin (HSA) and the receptor binding amino-terminal fragment (ATF) of uPA (Apparailly et al., 2002). Although speculative, it is possible that the inhibitory properties of mR1 is inadequate for such long-term treatment studies. The inhibitory mechanism of mR1 is unique in the sense that it traps a partially open conformation of unoccupied uPAR by binding to DI, but in the presence of elevated concentrations of uPA a ternary complex will form and this will drive uPAR into the closed conformation and thereby expel the bound mR1 (Gardsvoll et al., 2011a). The reverse scenario, where mR1 displaces uPAR-bound uPA, is not an option, since mR1 does not recognize the closed conformation of uPAR in the uPA•uPAR complex (Pass et al., 2007).

## *In vivo* Targeting of uPAR in Non-Invasive Imaging Modalities

The pronounced expression of uPAR at the invasive tumor-stroma microenvironment of most solid human cancers along with the correlation of high uPAR levels with poor patient prognosis, renders uPAR an attractive target for treatment modalities in aggressive cancers (Romer et al., 2004). Initially, such intervention programs primarily focused on function inhibition to dampen surface associated plasminogen activation (Crowley et al., 1993; Schmiedeberg et al., 2002; Lin et al., 2020; Yuan et al., 2021), but with the limited expression of uPAR in vital tissues, the focus gradually shifted toward targeted interventions based on cytotoxic eradication of uPAR expressing cells. Such uPAR-targeted treatment modalities include (i) recruiting the immune response to eliminate uPAR expressing cells using CAR-T cells (Amor et al., 2020) or priming the adaptive immune response with uPAR-targeted haptens (Rullo et al., 2016), (ii) proteolytic activation of prodrugs by uPAR-bound uPA (Liu et al., 2003; Gerspach et al., 2006; Schafer et al., 2011), (iii) uPAR-mediated internalization of cytotoxin-conjugated uPA-derivatives (Waldron et al., 2012; Zuppone et al., 2020) or antibodies (Harel et al., 2019), and (iv) targeted radiotherapy (Knör et al., 2008; Persson et al., 2012c; LeBeau et al., 2013). Notwithstanding the low uPAR expression in most vital tissues, the baseline expression in the glomeruli of normal kidneys may, however, pose a concern for such cytotoxic treatment modalities.

### Targeting uPAR With Peptide Antagonists

Although a wide array of intervention strategies based on uPAR-targeting have been designed and tested in preclinical animal studies, we will in the next sections primarily focus on studies using one particular 9-mer antagonist peptide denoted AE105 (Figures 1D,E) that binds human uPAR with high affinity. A 15-mer precursor peptide of AE105 was originally selected in an unbiased phage-display library and it bound uPAR with a K_D_ of 10 nM (Goodson et al., 1994). Using a functional scanning strategy, this 15-mer peptide was subsequently truncated to a decamer without substantial loss of affinity (Ploug, 1998; Ploug et al., 1998a). That truncated variant was subsequently used as template for the generation of focused combinatorial chemical bead-libraries with a view to affinity maturation and improvement in the biological stability of the peptide (Ploug et al., 2001). Panning these libraries with purified uPAR identified the 9-mer lead peptide (AE105), which bound uPAR with a K_D_ of 7 nM and exhibited a remarkable serum stability due its composition of both (L)-, (D)-, and non-natural amino acids (Figure 1D; Ploug et al., 2001). Derivatives of this 9-mer peptide was instrumental for solving the first crystal structure of human uPAR (Figure 1E; Llinas et al., 2005) and proved their diligence as the highly efficient uPAR-targeting core of several imaging probes used for non-invasive imaging of uPAR expression in patients with solid cancers (Ploug, 2013; Persson et al., 2015). The versatile applications of AE105 as uPAR-targeting moiety is illustrated in Table 1.

**TABLE 1:** Versatility in the applications of the uPAR targeting peptide AE105.

Compound	Reporter	Application	Model system	References

Non-invasive radionuclide imaging of uPAR expression				
[Cu^2+^]-DOTA–AE105 [Cu^2+^]-DOTA–AE105 [Cu^2+^]-CB-TE2A–AE105 [Cu^2+^]-CB-TE2A-PA–AE105 [Cu^2+^]-CHS1–AE105 [Cu^2+^]-DOTA–AE105	^64^Cu (PET) ^64^Cu (PET) ^64^Cu (PET) ^64^Cu (PET) ^64^Cu & Cy5.5 ^64^Cu (PET)	Preclinical Preclinical Preclinical Preclinical Preclinical Phase-1 trial	U87MG, MDA-MB-435 H727, U87MG, HT29 U87MG U87MG U87MG breast & prostate	Li et al., 2008 Persson et al., 2012a Persson et al., 2013a Persson et al., 2013a Sun et al., 2015 Persson et al., 2015
[Ga^3+^]-DOTA–AE105 [Ga^3+^]-NODAGA–AE105 [Ga^3+^]-NOTA–AE105 [Ga^3+^]-NOTA–AE105 [Ga^3+^]-NOTA–AE105	^68^Ga (PET) ^68^Ga (PET) ^68^Ga (PET) ^68^Ga (PET) ^68^Ga (PET)	Preclinical Preclinical Orthotopic Phase-1 trial Phase-2 trial	U87MG U87MG glioblastoma PDX breast, prostate, bladder prostate	[Bibr B136][Bibr B136]; [Bibr B186][Bibr B137][Bibr B170]; Fosbol et al., 2021a, b
[AlF]-NOTA–AE105	^18^F (PET)	Preclinical	PC-3	Persson et al., 2013b
[Tc^2+^]-DPA–AE105	^99*m*^Tc (SPECT)	Preclinical	PC-3	Kasten et al., 2016
[In^3+^]-DOTA–(AE105)_2_	^111^In (SPECT)	Preclinical	MDA-MB-231	Liu et al., 2009

**Fluorescence-guided optical imaging of uPAR expression**				

ICG-linker^1^–AE105 ICG-linker^1^–AE105 ICG-linker^1^–AE105 CH1055-linker^2^–AE105 IRDye800CW-linker^3^–AE105	ICG ICG ICG CH1055 IRDye800CW	Preclinical Orthotopic Orthotopic Orthotopic Orthotopic	U87MG OSC-19-*luc2* BxPC3-*luc2* U87MG U87MG	Juhl et al., 2016 Christensen et al., 2017 Juhl et al., 2019 Kurbegovic et al., 2018 Kurbegovic et al., 2021

**Targeted radiotherapy toward uPAR expressing cells**				

[Lu^2+^]-DOTA–AE105 [Bi^3+^]-DOTA–(linker^4^-AE105)_2_	^177^Lu ^213^Bi	Preclinical Preclinical	HT29, BxPC3-*luc2* OV-MZ-6	Persson et al., 2012c, [Bibr B133][Bibr B89]

**uPAR-targeting of nanoparticles for drug delivery**				

Dansyl-linker^5^–AE105 AE105 Cu2_x-RB@DMSN–AE105 AuNRs@SiO_2_–AE105 GNS@Ir@P–AE105	INOP INOP DMSN AuNRs@SiO_2_ Photoacustic	Cell culture Cell culture Preclinical Preclinical Preclinical	HEK293–uPAR cells PC-3 cells OSC-19 HeLa MDA-MD-231	Hansen et al., 2013 Ahmed et al., 2017 Zuo et al., 2020 Hu et al., 2019 Yu et al., 2020

**Miscellaneous applications of the uPAR-targeting peptide**				

Sepharose–(AE105)_2_ FAM–AE147 (AE105)_2_ AE147	Sepharose Fluorescein None Hg modification	Purification FP-assay ELISA Structure	Cell culture media Small molecule inhbitors Human plasma samples uPAR•AE147 crystals	Jacobsen et al., 2007 Mani et al., 2013 Piironen et al., 2004 Llinas et al., 2005

*AuNRs@SiO_2_, mesoporous silica-coated Au nanorods; CB-TE2A, 4,11-bis(carboxymethyl)-1,4,8,11-tetraazabicyclo[6.6.2]hexadecane; CB-TE2A-PA, 4-carboxymethyl-11-(1,3-dicarboxypropyl)-1,4,8,11-tetraazabicyclo[6.6.2]hexadecane; DMSN, dendritic mesoporous silica nanoparticles; DOTA, 1,4,7,10-tetraazadodecane-N,N’,N”,N”’-tetraacetic acid; DPA:2,2’, dipicolylamine; ICG, indocyanine green; INOP, ion oxide nanoparticle; Linker^1^, EE; Linker^2^, EEEE; Linker^3^, EE(OEG)_2_; Linker^4^, KGSGG; Linker^5^, KGSGSGG; NODAGA, 2-(4,7-bis(carboxymethyl)-1,4,7-triazonan-1-yl)pentanedioic acid; NOTA, 1,4,7-triazacyclononane-1,4,7-triacetic acid; PDX, patient derived xenograft.*

#### Positron Emission Tomography

**FIGURE 3:**
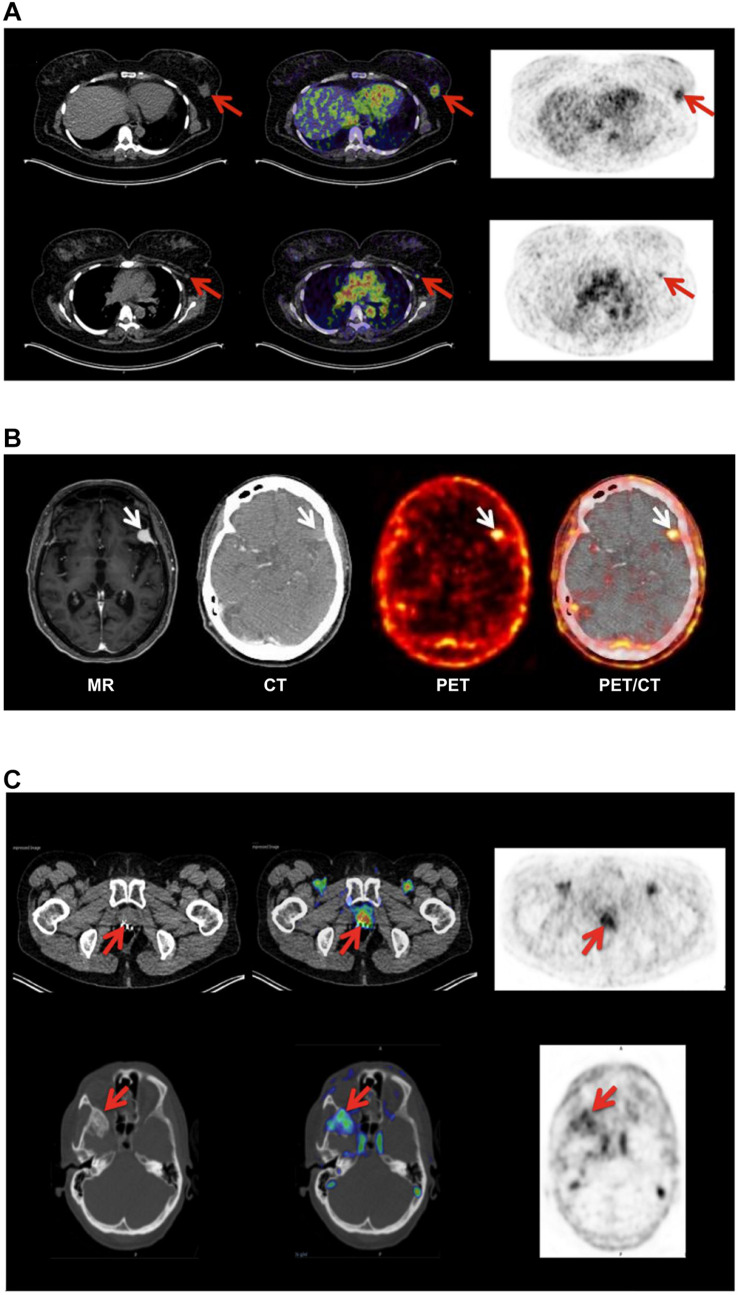
Non-invasive PET imaging of uPAR expression with AE105 as targeting principle. **(A)** Non-invasive PET/CT imaging of a breast cancer patient 10–60 min post *iv* injection of ^68^Ga-NOTA–AE105. Shown to the left are the CT scans, to the right PET scans, and in the middle the merged images. The red arrows highlight (i) the uPAR-positive primary breast cancer lesion (upper row) and (ii) an uPAR-positive axillary lymph node lesion (lower row) that escaped detection by preoperative ultrasound scanning and fine-needle biopsy. **(B)** Non-invasive PET/CT image of the transversal cross-section of the brain of another breast cancer patient with confirmed primary breast cancer lesion 60 min post *iv* injection of ^64^Cu-DOTA–AE105. High tracer uptake was unexpectedly noted in a brain lesion that was later confirmed by magnetic resonance scanning (MR) and diagnosed as a meningioma (white arrow). **(C)** PET/CT imaging of a prostate cancer patient using ^68^Ga-NOTA–AE105 to visualize uPAR expression. Red arrows highlight tracer accumulation in the primary prostate lesion (upper row) and in a metastasis lodged in the sphenoid bone (lower row). Images were reproduced from Persson et al. (2015) and Skovgaard et al. (2017) under the CC-BY license.

Position emission tomography (PET) combined with computed tomography (CT) constitute the central imaging platform in clinical oncology (Duclos et al., 2021). The overarching virtue of PET/CT is that it is (i) non-invasive, (ii) provides an overview of the global expression of a given molecular target or function shortly after injection of the radionuclide tracer, (iii) has no issues with sampling bias, and (iv) is highly versatile in the sense that the collection of molecular targets that can be visualized depends only on the availability of a suitable PET probe. Although ^18^F-fluorodeoxyglucose (^18^F-FDG) is the unopposed toiler of PET imaging in nuclear medicine, new peptide-based PET probes are continuously being developed thus widening the range of molecular targets that can be visualized (Askari Rizvi and Zhang, 2021). As alluded to in the previous sections, the increased uPAR-expression in the tumor-stromal microenvironment of invading cancer lesions makes it an attractive target for molecular imaging in the clinical assessment of tumor invasion and metastatic dissemination. As listed in Table 1, a plethora of PET-probes, targeting uPAR *via* the AE105 moiety, have been designed and explored in preclinical animal models bearing various human cancers (subcutaneous, orthotopic, and patient derived xenografts). These studies include xenografts from different origins (e.g., brain, prostate, lung, and breast) and use PET tracers combining various macrocyclic chelators (DOTA, NOTA, CB-TE2, CB-TE2A-PA) with different radionuclides (^64^Cu, ^68^Ga, Al^19^F) - as shown in Table 1 (Price and Orvig, 2014). All studies showed specific uptake in tumor lesions and the uptake levels correlated to uPAR levels as determined by ELISA or immunohistochemistry in resected subcutaneous tumor xenografts (Persson et al., 2012a, 2013a). It should be emphasized that the endogenous expression of mouse uPAR will not be detected in these imaging studies as the AE105 targeting moiety is strictly specific for human uPAR (Ploug et al., 2001). Building on these encouraging preclinical mouse studies, the first phase-1 clinical trials were conducted in human cancer patients with ^64^Cu-DOTA–AE105 (Persson et al., 2015) and ^68^Ga-NOTA–AE105 (Skovgaard et al., 2017). Both studies showed no adverse pharmacological effects and internal radiation burdens were equivalent to that of standard ^19^F-FDG PET scans. Importantly, specific tracer uptake was noted in primary lesions as well as in several lymph node metastases and subsequent immunohistochemistry on resected tumor tissue confirmed that all these lesions were indeed uPAR positive. Circumstantial observations from these phase-1 clinical trials demonstrate the power of such global and unbiased uPAR–PET imaging (Figure 3). First, one patient enrolled with an indication of breast cancer revealed an unexpected additional tracer uptake in the brain, which was later diagnosed as a primary meningioma (Persson et al., 2015). Second, another breast cancer patient had a robust tracer uptake in two axillary lymph nodes despite previous preoperative staging by ultrasound, fine-needle biopsy, and CT failed to detect any malignant lymph node involvement (Skovgaard et al., 2017). A small prospective phase-2 clinical trial on ^68^Ga-NOTA–AE105 uptake in the primary prostate lesions of 27 cancer patients found a positive correlation between standard uptake values (SUV_max_) and Gleason score based on histopathological grading of biopsy specimens (Fosbol et al., 2021a). In a small follow-up study, baseline SUV_max_ of ^68^Ga-NOTA–AE105 in bone metastasis index lesions of 14 prostate cancer patients were measured before two cycles of ^223^RaCl_2_ therapy. Despite the low number of patients enrolled in this study, baseline SUV_max_ correlated to overall survival (Fosbol et al., 2021b). Notwithstanding these promising results, large scale clinical trials are needed to precisely define the clinical utility of uPAR-PET scans in cancer patient management.

Due to the short half-lives of traditional positron emitting radionuclides [^68^Ga (67.7 min), ^18^F (109.7 min), and ^64^Cu (12.7 h)], the majority of PET probes rely on small molecules or peptides as their targeting moiety due a fast pharmacokinetic profile with rapid contrast development. The clinical implementation of immunotherapy created, however, an unmet need for immuno-PET imaging to stratify and select patients eligible for antibody-based therapy (Liberini et al., 2021). To create a better match with the slow pharmacokinetics of antibodies, most immuno-PET studies use ^89^Zr as positron emitting nuclide with a half-life of 3.3 days. So far this technology has not been applied to uPAR-PET imaging, but was used to image its high-affinity protease ligand uPA in subcutaneous tumors using an ^89^Zr-labeled mouse monoclonal antibody (ATN291) that is specific for human uPA (Yang et al., 2016). It is unclear whether this imaging represents uPA in complex with uPAR on the cells surface of the transplanted human cancer cells. Two different laboratories conducted low resolution imaging with single photon emission computed tomography (SPECT) to visualize uPAR expression in xenografts using the ^111^In-labeled monoclonal anti-huPAR antibodies ATN658 (Boonstra et al., 2015, 2017) and 2G10 (LeBeau et al., 2013, 2014). Both approaches provided clear primary tumor delineations 72 h post tracer *iv* injection and ^111^In-labeled 2G10 detected occult bone metastasis in mice with triple negative human breast cancer xenografts (LeBeau et al., 2013).

#### Fluorescence-Guided Intraoperative Imaging

The Holy Grail in cancer surgery is the radical removal of all malignant tissue creating tumor free resection margins while preserving as much of the healthy tissue as possible—ultimately leading to improvements in progression-free survival. While PET/CT and MRI imaging platforms are important for staging and localization of disseminated disease, they cannot easily guide surgical procedures in real-time. In the last decade, design of targeted near-infrared (NIR) probes for fluorescence-guided cancer surgery enabled the rapid development of emerging imaging tools that could potentially assist surgical navigation in real-time (Debie and Hernot, 2019). Although still in its infancy, targeted imaging in fluorescence-guided cancer surgery is gaining momentum especially with antibodies that are already applied in therapy or have a strong biomarker potential. But proof of concept studies demonstrating the added beneficial effects for cancer patient survival are still lacking (Hernot et al., 2019).

**FIGURE 4:**
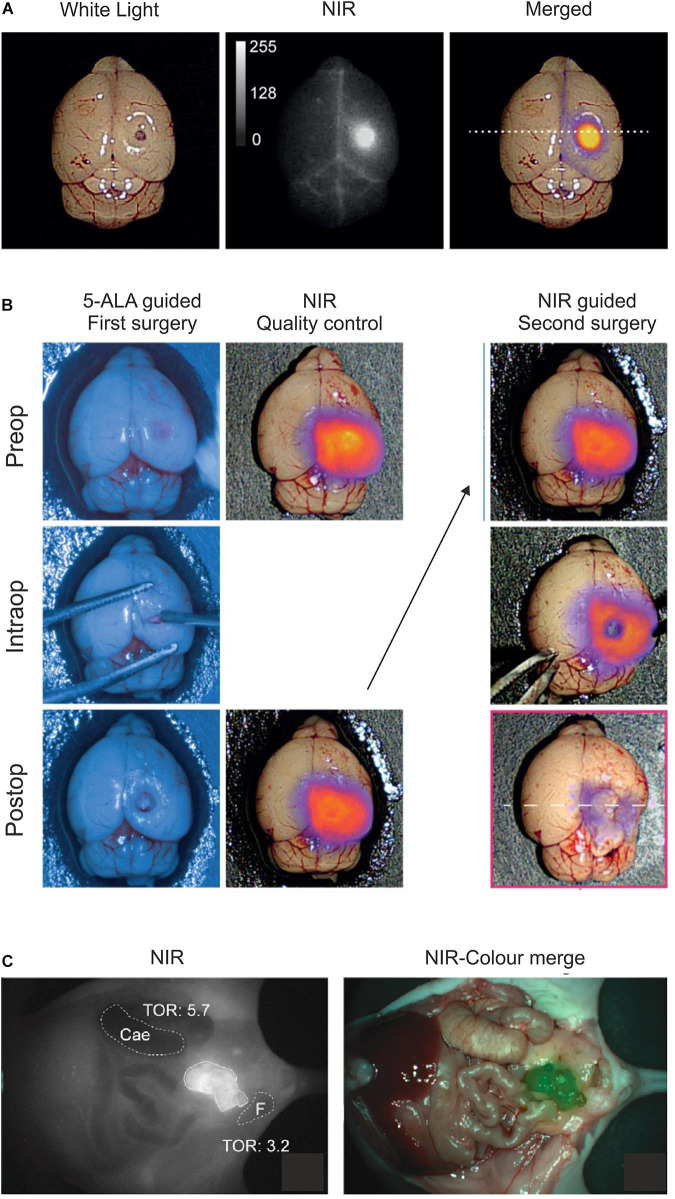
Optical imaging of uPAR expression with AE105 as targeting principle in mouse models. **(A)** Intact resected mouse brain with an orthotropic xenotransplant of U87MG cells imaged 1.5 h after *iv* injection of 6 nmol IRDye800CW-linker^3^–AE105. *Left panel* shows image with white light; *middle panel* shows NIR image; and *right panel* shows the merged image using pseudo-colors to illustrate relative tracer uptake—low to high uptake: blue→red→yellow. **(B)** Fluorescence guided intraoperative imaging following the surgical resection of an orthotropic U87MG transplant. *Left panel* shows the initial surgical brain resection guided by the real-time fluorescence signal from protoporphyrin IX after 5-aminolevulinic acid administration (5-ALA, Gliolan^®^); *middle panel* shows the corresponding NIR control images from IRDye800CW-linker^3^–AE105 fluorescence of the surgical bed before and after resection guided by 5-ALA; and the *right panel* shows the subsequent surgical resection guided by NIR fluorescence and visualized by a modified EleVision^TM^IR system (Medtronic, MN, United States). **(C)** NIR and merged images recorded 3 days post *iv* injection of 1 nmol IRDye800CW–ATN658 (a humanized monoclonal anti-uPAR antibody) in a orthotropic mouse model of urothelial cell carcinoma. **(A,B)** were reproduced and modified from Kurbegovic et al. (2021) and **(C)** from Baart et al. (2021) according to the CC-BY license.

As alluded to in the previous sections, baseline expression of uPAR is low and scattered in normal healthy tissues while it is robustly upregulated in most active cancer lesions, particularly in the microenvironment of the invading tumor-stromal interface. This expression pattern makes uPAR an ideal target candidate in fluorescence guided intraoperative imaging and several strategies have accordingly been explored in preclinical mouse models with AE105 as the targeting core (Table 1). Using NIR fluorophores as reporter groups increases the depth of the surgical field that can be visualized due to a higher tissue penetration and a decreased auto-fluorescence of wavelengths in the NIR-I (650–900 nm) and NIR-II (1,000–1,700 nm) window. The first generation of uPAR-specific optical probes exploited the clinically approved fluorophore indocyanine green (ICG) as reporter group (Juhl et al., 2016). Although the conjugation of ICG to AE105 reduced the affinity for uPAR by 20-fold, initial optical imaging studies on subcutaneous xenotransplants of the U87MG glioblastoma cell line (Allen et al., 2016) led to a specific probe uptake reaching maximal tumor-to-background ratio (TBR) after 6–24 h post *iv* administration (Juhl et al., 2016). The ICG–AE105 conjugate also showed promise in the real-time guidance of preclinical surgery of xenotransplanted orthotropic mouse models of head-and-neck and pancreatic cancers (Christensen et al., 2017; Juhl et al., 2019). Building on these promising preclinical studies, a phase 1–2 clinical trial was launched in 2020 exploring the safety and use of ICG–AE105 in patients with glioblastoma (clinicaltrialsregister.eu, EudraCT 2020-003089-38). In second generation AE105-conjugates, ICG was replaced by fluorophores with more optimal spectral properties i.e., CH1055 (NIR-II) and IRDye800CW (Kurbegovic et al., 2018, 2021). Especially IRDye800CW–AE105 was optimized biochemically and analyzed extensively in a surrogate glioblastoma mouse model with U89MG cells as orthotropic transplants (Figures 4A,B) (Kurbegovic et al., 2021). Testing different linker sequences, the IRDye800CW-linker^3^–AE105 conjugate was selected (Table 1), which binds uPAR with only 3-fold lower affinity as compared to unconjugated AE105. Dependent on the amount of probe administered, maximal TBRs of 6.6 to 7.0 were reached within 1–3 h post *iv* injection. The fast kinetics in generating optimal contrast is remarkable and is most likely a consequence of the relatively small size and hydrophilicity of this probe as compared to ICG-AE105 (more hydrophobic) and the larger antibody based probes, where optimal contrast for the latter typically requires a much longer probe washout (3–5 days).

Besides peptide-based targeting of uPAR—the topic of this review—several macromolecular uPAR-targeting moieties have been developed for optical imaging and therapeutic targeting including (i) monoclonal anti-uPAR antibodies (Figure 4C; LeBeau et al., 2014; Boonstra et al., 2015; Baart et al., 2021) and (ii) various nanoparticles carrying receptor binding fragments of uPA (ATF) or peptide derivatives (AE105) (Hansen et al., 2007; Yang et al., 2013; Gao et al., 2017; Zuo et al., 2020). Due to the inherently long “washout times” (days rather than hours) needed for these probes to reach maximal TBR, they are probably less suited in the daily clinical workflow as imaging modalities, but they are optimally suited for targeted delivery of cytotoxic payloads.

#### Targeting uPAR With Therapeutic Intention

In the last two decades, a variety of different targeted intervention strategies have been developed to eradicate uPAR-expressing cells. Specificity of the cytotoxic insult were devised by targeting uPAR directly or by exploiting the proteolytic activity of uPAR-bound uPA (Liu et al., 2003; Rustamzadeh et al., 2007; Schafer et al., 2011; Morodomi et al., 2012; LeBeau et al., 2013; Jing et al., 2017; Harel et al., 2019; Amor et al., 2020; Zuppone et al., 2020). Several of these treatment modalities have shown promising results in preclinical mouse models bearing human cancer cell xenografts. One small study showed beneficial effects on canine oral mucosal melanomas inducing stable disease in all five dogs treated with uPA- and MMP2-activated anthrax toxin (Nishiya et al., 2020). But none of these modalities have so far entered clinical trials in humans. We predict that the non-invasive uPAR–PET imaging platforms discussed in this review will greatly facilitate the future clinical translation of a given uPAR-targeted therapy regimen—assisting in both initial patient selection based of target availability as well as response marker monitoring treatment efficacy.

## Conclusion

Research performed in the last two decades have provided a detailed outline for the structure-function relationships in components responsible for cell surface associated plasminogen activation. This knowledge has been instrumental for designing a plethora of intervention strategies to target these components or their function with special emphasis on treatment modalities in oncology. Opportunities to target uPAR in inflammatory diseases beyond cancer should, however, also be considered (Baart et al., 2020).

## Author Contributions

JL and MP wrote the manuscript. Both authors contributed to the article and approved the submitted version.

## Conflict of Interest

The authors declare that the research was conducted in the absence of any commercial or financial relationships that could be construed as a potential conflict of interest.

## Publisher’s Note

All claims expressed in this article are solely those of the authors and do not necessarily represent those of their affiliated organizations, or those of the publisher, the editors and the reviewers. Any product that may be evaluated in this article, or claim that may be made by its manufacturer, is not guaranteed or endorsed by the publisher.

## Footnotes

1 ATF, amino-terminal fragment of uPA; DI-DII-DIII, the first, second and third LU domain in uPAR; GFD, growth factor -like domain; GPI, glycosyl-phosphatidylinositol; ICG, indocyanine green; LU, Ly6/uPAR; NIR, near-infrared; PET, positron emission tomography; PNH, paroxysmal nocturnal hemoglobinuria; SMB, somatomedin B; tPA, tissue-type plasminogen activator; uPA, urokinase-type plasminogen activator; uPAR, uPA receptor.

2 A baseline concentration of 20 pM prouPA—slightly below or equal to the K_D_ for its interaction with uPAR—makes this interaction optimally suited to sense and respond to variations in prouPA concentrations during e.g., chronic inflammation.

3 *Plau*^*GFDhu*^ is the mouse gene encoding uPA where the key residues for binding to mouse uPAR (Tyr^23^, Arg^28^, Arg^30^, and Arg^31^) were replaced by the corresponding residues from human uPA (Asn^22^, Asn^27^, His^29^, and Trp^30^), which leads to an 400-fold decrease in the affinity for mouse uPAR (Connolly et al., 2010; Lin et al., 2010) thus essentially uncoupling that reaction.
